# From legends to legacy: the impact of fan influence on retiring athletes in premier league basketball

**DOI:** 10.3389/fpsyg.2023.1295487

**Published:** 2023-11-08

**Authors:** Assaf Lev, Orr Levental, Ilan Tamir

**Affiliations:** ^1^Department of Sports Therapy, Faculty of Health Professions, Ono Academic College, Kiryat Ono, Israel; ^2^Department of Physical Education, Tel Hai College, Tel Hai, Israel; ^3^Moskowitz School of Communication, Ariel University, Ariel, Israel

**Keywords:** basketball, retirement, age, fandom, qualitative study, body, media, Israel

## Abstract

**Introduction:**

This research examines the perceived age of Premier League basketball players as they near retirement, focusing on the complex interplay between players, fans, and the media in shaping perceptions of age and retirement. The study highlights the unique pressure on the basketball players to retire due to age-related expectations, rooted in the perception that athleticism is age dependent.

**Methods:**

The research methodology applied in this study involved conducting semi-structured interviews with a sample of 11 professional Israeli basketball players and two veteran players’ agents.

**Results:**

Three themes emerged: (1) Feelings of betrayal and ingratitude; (2) A farewell tour toward disengagement from one’s professional career; and (3) The media’s role in shaping beliefs and attitudes. These themes illustrate how the sports environment portrays aging players, shaping fan opinions, both positively and negatively. Significantly, the findings emphasize the challenges that players must confront within an ageist environment.

**Discussion:**

The study concludes by highlighting the cultural and social construction at work. The “coercion” to retire from professional sports is more pronounced here due to the common belief that sports success is time-bound and age-dependent, necessitating a “young body”. In this reality, the audience does not merely function as a backdrop for athletes’ performances, but has the power, at the collective and the individual level, to affect change on and off the court.

## Introduction

The age of athletes plays a critical role in shaping not only how they are perceived by others but also in influencing their self-perception. This becomes increasingly evident as athletes approach retirement, their age becoming a central theme in both public discourse and professional discussions. One of the most pivotal aspects in this context is the dynamic relationship between fans and athletes. This relationship is heavily influenced by how fans perceive an athlete’s skills, expectations, and behaviors. As highlighted by Cosh et al. (2013), athletes typically retire at a relatively young age, a phenomenon often driven by the prevailing norms within specific sports. Consequently, age emerges as a primary determinant of retirement, frequently limiting an athlete’s autonomy in determining their career trajectory. Understanding the roles played by various social agents in the realm of sports, as well as the experiences of athletes during the latter stages of their careers, carries both theoretical and practical implications. Despite its prevalence and significance, this particular aspect, which forms the central focus of the present study, has been largely underexplored in academic research. Consequently, this study aims to fill this gap by providing a comprehensive examination of how basketball players perceive the treatment they receive from both fans and the media.

### Sports fans

Sports fandom is an exceptionally widespread, cross-national and cross-cultural phenomenon, considered unique in many respects, and has therefore captured extensive research attention in recent years. The fundamental presumption, which constitutes a solid foundation for research centered on sports fans’ behaviors and speech, is that sports function as authorization zones for fans (Ben-Porat, 2014), granting fans the legitimacy to behave freely and spontaneously in ways that are unacceptable outside the stadium. Therefore, an analysis of fans’ behaviors or discourse can reach beyond the politically correct dialog that characterizes many societies (Sorek, 2019) and capture authentic thoughts, attitudes, and opinions. The ethical perspective that is acceptable in the world of sports is that fans occasionally have additional rights related to the negative ways in which they express their fandom, such as curses, insults, and jeering directed to the rival team and even to its players (Smith and Lord, 2018). According to Marmo (2012), such communications between fans and players not only affect players’ performances but also their cognitive functioning. Extending beyond elevating or reducing players’ motivation, the audience also affects players’ attitudes and perceptions, including their self-concept, as discussed below. For sports fans, their team is an important component of their lives and their fundamental identity (Tamir, 2022). Fans will accompany their team in all circumstances and to almost all places, making enormous sacrifices for the team, even going so far as to sacrifice their health for the team (Levental et al., 2021). In addition to encouragement and support, fandom practices may also manifest as violent behavior and badgering players and rival fans (Duncan, 2019). At the same time, fans might lavish compliments on certain players and mark players as heroes based on their traits and behaviors (Levental and Nudelman, 2021). Thus, the audience does not serve merely as a backdrop to players’ performances, but actually affects diverse elements and actions that occur in the competition sphere, at both the group level (Unkelbach and Memmert, 2010) and the individual level (Marmo, 2012). The impact on players also depends on the players themselves, but fans may directly affect a player’s self-image and, consequently, the player’s professional performance (Heaton and Sigall, 1991). Fans’ interest in and commitment to their team also drives a hunger for information related to the team and its players, and anything surrounding them (Aiken et al., 2018). As a result, sports media have become one of the most popular, significant, and influence sectors of the media industry (Tamir and Lehman-Wilzig, 2023). It is therefore unsurprising that studies have identified media consumption’s effects on sports fans, on various levels, from basic information to fans’ identity and identification with a team (Phua, 2010).

Media framing theory is considered one of the most well-known and influential theories in the study of media effects. The theory deals with the question of how the media mediates reality to the audience. The premise is that the media creates the nature of the discourse, and public opinion absorbs and internalizes it. Framing is defined as the way in which a media source defines, organizes, and structures the media information, so that the sequence of events covered receives broad context and meaning (Gamson and Modigliani, 1987). Framing can be done intentionally and with the knowledge of the addresser, but it can also be done subconsciously (Gamson, 1989). But the basic assumption is that in any press coverage, the media applies certain media frames to the happenings. According to Entman (1993), the act of framing includes four functions: (a) defining a problem (b) diagnosing the causes (c) moral judgment (d) calling for action (proposing a solution). And so, the frame becomes an interpretive scheme that affects the reader and allows him to identify, label, process the events and store the information for the long term. Media frames are constructs that align with the preexisting mental frameworks of public opinion to the extent that the audience comprehends the cues presented in the media effortlessly, often without conscious awareness (Entman, 2004). In the particular context of older athletes, whether conveyed through traditional communication channels or within the discourse of new media, including social networks, the coverage on aging athletes frequently accentuates their age, effectively framing the narrative around this aspect (Carmack and Lazenby, 2023). In this respect, sports media’s framing of complex issues related to a sports player’s identity, and to sports in general, has a strong impact on media consumers’ social perceptions (van Sterkenburg et al., 2010).

According to Hasaan et al. (2016), mass media and interpersonal communications are key components in the construction of an athlete’s personal brand. These media are the primary means for shaping fans’ perceptions about certain players and therefore have a broad effect on a player’s public image and self-image. In turn, a player’s image directs fans’ attitudes toward a player and their consumption behaviors in general (Vieira and Sousa, 2020). As Arai et al. (2014) note, the athlete’s brand is influenced by their on-field performance and external factors like their appearance and conduct outside the sporting arena. However, this branding is dynamic as it is directly influenced by perceived factors such as successful performance on the court and, to a large extent, by differences in the player’s career stage (Hodge and Walker, 2015). It should be noted that over the past decade, there has been a notable surge in the utilization of social media as a tool for bidirectional communication between athletes and their fans, particularly in response to the COVID-19 pandemic (Su et al., 2020). Effectively using social media can influence players’ engagement with both fans and teammates, contributing positively to a player’s branding (Doyle et al., 2022). However, the present study aims to concentrate on the immediate and unidirectional relationship within sports arenas and the combined impacts of the audience stemming from their behavior.

The genesis of Israeli sports and the affiliation of fans to their beloved clubs were initially rooted in deep political involvement (Galily, 2007). However, trends of commercialization and professionalization, especially during the 1980s, led to a distancing from ideological affiliations and to the emergence of a global fan culture (Ben-Porat, 2000). Despite the processes of sports fandom globalization, there are still cultural components that manifest, including militarism prevalent in Israeli society, entering the realm of fan culture (Roth-Cohen and Tamir, 2017), as well as idealized perceptions of athletes (Levental et al., 2022). These factors do indeed impact fans’ expectations of players but are not fundamentally different from the branding processes undergone by top athletes worldwide. Therefore, the current research discussion in the Israeli context is not necessarily shaped solely by the local context but describes a commonly accepted dynamic between crowds and players during games.

### Athletes, age(ing), and retirement

It is common knowledge that a professional sports career is both contingent on and limited by one’s biological age, more so than in any other profession. While considerations of retirement in other professions are not necessarily considered to entail one’s body, retirement from professional sports is intuitively considered a “natural” process that occurs at relatively young ages.

Webb et al. (1998) argued that athletes have two types of sports identities: a private identity, which refers mainly to an athlete’s personal feelings and emotions, including their self-image, and a public identity, which refers to the ways in which the athletes perceive their popularity and public admiration during their professional career. A study on professional athletes’ identity and retirement illustrates this distinction (Lally, 2007). Lally argued that in anticipation of their transition to life after a professional career, athletes begin to negotiate “a redefinition of the self” (p. 12). The external aspects of body presentation and age, which mediate between the individual and their social identity as they approach retirement, are largely shaped by the athlete’s efforts at impression management (Lev and Weinish, 2020; Weinish and Lev, 2021) in advance of their retirement. According to Goffman (1959), both the individual and the environment participate in socially constructed patterns, in which they are mutually recognized and assessed, and positioned in social contexts and power structures. In circumstances where elite athletes may experience a drastic decline in their self-image and competitive abilities after retirement (Stephan, 2003), they must be vigilant about the external performance dimensions as a means of self-presentation to others. Relatedly, Jones and Denison (2017), who studied soccer players who had retired from England’s premier league, discovered that the retirement process was not only a challenge but also a source of relief from their need to manage their body. Age as a stratified element thus become a major factor in the course of a professional athlete’s career. In this context, the concept of ageism is often associated with terms such as “stereotypes,” “prejudices,” “stigma,” “racism,” and “discrimination” when discussing “old age” and “the elderly.” Most studies align with Butler’s (1969) definition of ageism, which characterized it as a process involving the stereotyping and systematic discrimination against individuals solely due to their advanced age. In a more contemporary interpretation, ageism is defined as a complex amalgamation of various facets, including a sociological assessment of unequal distribution of resources and access to them, heightened awareness of the emotional consequences of this exclusion, societal critique of biases and discriminatory patterns, and the disadvantaged legal status often experienced by elderly individuals (Hazan, 2013). More specifically regarding athletes, it therefore appears that of a wide spectrum of professions, “athletes constitute an extreme example of this, as their physical ability reaches a competitive peak at an extremely early chronological age” (Hazan, 1984).

Kama and First (2015) argued that older populations inescapably become a powerless group characterized by a negative image, and experience symbolic extinction and disregard on the part of the media. Although in their study they refer to adults at the end of their lives, while this study focuses on young people at the end of their professional career, it is notable that attitudes toward professional basketball players approaching retirement is similar to the treatment that older adults receive.

According to the stereotype embodiment theory, people internalize socially generated stereotypes about aging into their perceptions of their own aging which can lead to self-fulfilling prophecies that have a negative impact on their health (Levy, 2009; Silver, 2021). For athletes, retirement is considered to be a critical stage in one’s career, and has accordingly attracted research attention. Considering athletes’ extensive public exposure and attention, it is understandable that retirement is accompanied by multiple social, physical, and mental challenges and difficulties and benefits (Jewett et al., 2019). Wippert and Wippert (2008) reported a significant and systematic decline in athletes’ self-reported stress immediately after retirement.

Mihovilovic’s (1968) pioneering study adeptly described the difficulties and anguish before retirement from professional sports, but studies show that retirement is perceived to be much less problematic when it is planned as a process rather than an event (Torregrosa et al., 2004), and that pre-retirement planning that takes into account the psychological, financial, and other implications of retirement significantly contributes to the quality of the transition and to athletes’ adjustment to their post-career status (Warriner and Lavallee, 2008). Moreover, a redefinition of the self, through proactive diminishment of one’s pre-retirement athletic image, can be very beneficial in this process. Nonetheless, in many cases, retirement is accompanied by a sense of confusion and loss of identity, which may persist for many years after retirement (Kerr and Dacyshyn, 2000). However, it should be stressed that these factors vary from one cultural context to another, where characteristics of specific social contexts can serve as a resource or barrier to athletes in advanced career stages (Alfermann et al., 2004).

The current study focuses on the specific social context of basketball players in Israel and explores the audiences’ role in professional basketball players’ retirement process.

## Methodology

### Participants

The study’s objective is to gain an understanding of how Premier League basketball players perceive age as they near retirement and to explore the role played by their fan base in this transitional process. Eleven professional Israeli-born basketball players participated in the current study and two veteran basketball players’ agents. Their mean age ranged from 30 to 40 years and playing experience ranged from 14 to 22 years. The first six interviewees were recruited by the first author, a former professional basketball player in Israel’s First (Premier) League, who was then referred to an additional five participants (i.e., snowball sampling; Sparkes and Smith, 2013).

**Table 1:** 

Name	Age	Living area	Position
Nimrod	31	Tel Aviv	Player
Eran	32	Tel Aviv	Player
Shlomi	34	Tel Aviv	Player
Dennis	39	Tel Aviv	Player
Amit	32	Tel Aviv	Player
Ronnie	35	Tel Aviv	Player
Eli	33	Beer Sheva	Player
Haim	33	Northern Israel	Player
Elad	31	Northern Israel	Player
Orr	34	Northern Israel	Player
Oren	35	Northern Israel	Player
Michael	41	Tel Aviv	Agent
Yossi	43	Tel Aviv	Agent

### Instruments

Materials from the field were collected using semi-structured interviews. Semi-structured interviews were employed because they offered the interviewers a large degree of flexibility in posing spontaneous questions on how older basketball players perceive the late stage of their career. Moreover, semi-structured interviews are a vital source of rich knowledge and encourage participants to relay personally meaningful experiences in which they divulge noteworthy aspects of human behavior in everyday life (Brinkmann, 2013). The players were asked mainly about the way they perceive the treatment they receive from both fans and the media (e.g., How do you perceive the influence of fan expectations and reactions on basketball players’ decisions to retire or continue their careers? How does the pressure of meeting fan expectations affect players’ self-image as they approach the end of their basketball career? How do you perceive the role of media coverage and scrutiny in shaping players’ decisions to retire or prolong their career? How does media coverage and public opinion affect players’ self-image, motivation, and confidence as they approach the end of their basketball career?). The duration of each interview ranged from 45 to 75 min. Participants were selected on the basis of their relationship with the first author, and their willingness to serve as key informants and share their firsthand experiences. The questions for the semi-structured interview were designed by the first author. His background as a former professional basketball player in Israel’s Premier League provided him with a unique insider/outsider perspective. This perspective was invaluable in understanding how professional basketball players perceive age as they approach retirement and the influence of their fan base in this context. Prior to each interview, participants were informed about the study’s aims and methods. The interviews were conducted in Hebrew and translated into English by the researcher.

### Data analysis

The present study employed the thematic analysis method to comprehensively examine the data and identify prominent themes within a dataset. The purpose of this analysis was to elucidate and interpret the underlying meanings and significance of these themes (Braun et al., 2016). The analytical exploration of the data involved immersing in the transcripts, coding the data, and generating themes. An inductive approach was adopted, whereby themes and patterns emerged from the data itself. Additionally, the analysis engaged with the data on a latent level, focusing on underlying ideas, beliefs, and perspectives that were not explicitly expressed. While most thematic analyses encompass both latent and semantic coding (Braun et al., 2016), the latter was occasionally employed in this study to decode explicit concepts, ideas, or patterns directly evident in the data. Through the organization of data into higher-order themes, certain codes were examined and subsequently amalgamated to shape overarching themes. For instance, the code “Fan’s Behavior Outside the Arena” was integrated with the theme “Feelings of Betrayal and Ingratitude.” The following overarching themes were identified: A sense of betrayal and ingratitude, a farewell tour in anticipation of one’s disengagement from one’s professional career, and the media’s role in shaping the player’s image as they approach retirement. Our theme development process involved a continuous examination of fundamental inquiries: (1) Identifying the core essence of each theme, (2) assessing their meaningful contributions to both the dataset and our research questions, (3) establishing clear boundaries for each theme, and (4) ensuring thematic coherence. With these well-defined themes in hand, our attention turned to crafting integrated explanations across the overarching themes. This phase involved in-depth exploration of the relationships between themes, probing how they intertwined to construct a comprehensive and nuanced portrayal of the data. As these themes were described and contemplated, relevant literature was employed to establish connections between the themes and the societal experiences of the linkage between professional basketball players toward the end of their career and their relationship with fans, thus encompassing the findings.

### Ethical requirements and quality assurance

Several steps were taken to ensure the study’s compliance with ethical standards. First, approval was obtained from the institutional review board. Participants provided informed consent, using common pseudonyms to protect their identities and to clarify the gender of each participant for readers. To ensure quality and trustworthiness, Tracy’s (2010) eight hallmarks for high quality qualitative methods across paradigms were implemented. Relevant theoretical foundations were applied to achieve the study’s objectives, methodology, and findings. More specifically, the first author conducted iterative readings and deeply immersed himself in the transcripts. Following this, a collaborative approach was adopted to analyze the material and resolve any disparities with fellow researchers, resulting in the creation of a final, cohesive version of the coded notes. This procedure was iterated until a list of themes was supported by consensus to represent the data.

## Findings and discussion

### Feelings of betrayal and ingratitude

The first theme addresses the dialog between the players and their audience, and specifically the audience’s attitude to the players considered past their athletic prime. Because the player does not necessarily concur with this assessment, the player views the fans’ statements and behavior as unfair and in violation of the traditional function of fans, which is to support the team and its player in all circumstances (Tamir, 2022). From the player’s perspective, withholding support is equivalent to betrayal and reflects fans’ ungratefulness for the player’s investment of time and effort in the game and the team, and the player’s efforts for the fans. Nimrod, a 31-year-old basketball player, described his bitter experience with the fans during one game:

I was in what might be the most difficult situation in basketball... I faced 3,000 comments in the arena, shouts, curses: “What are you doing?!” “Go home!” “You’re a nothing!” from the supposed fans of your team... and it’s a very difficult experience… very difficult... especially for someone who is very self-critical and very sensitive about things like that. It was very, very difficult. These were sharp, personal remarks and insults.

Eran, a 32-year-old basketball player, also described the importance of a player’s approach to such retorts:

It [the audience’s remarks] really affected me… not necessarily just those about my age…. If, for example, they shout something like “Old man, retire,” then I will retire, you know? From time to time, they would call me “fatso” and stuff like that, you know how it is, they are always looking for something…. My whole life, if someone put me down, it only made me stronger, so then I go out there to prove it – here, I’ll beat you now…. Still, I say, when a guy is 31, 32, what do you want from him? I really do not get it, I do not get it, I told you – it’s a matter of attitude. It’s something that you cannot explain. I told you that the biggest problem is that maybe they are all right. After all, you see that you are still here, but you tell yourself, “maybe they are right and I really am finished.”

The taunts on the court are the main dimension in the process in which the players, their self-image, and their qualifications are affected and shaped by the audience (Marmo, 2012). Eran chose to deal with the offensive comments in a positive manner by constructively using them to motivate and drive himself. But this is no more than an optimistic approach to a process to which he is fated by his age. The audience’s taunts clearly affect Eran’s self-image. The words “maybe they are right and I really am finished” are indicative of the enormous power that the fans and spectators hold, and already enfold capitulation on Eran’s part. They also resonate with the stereotype embodiment theory which posits that stereotypes are embodied when they are absorbed from the surrounding environment, subsequently shaping self-definitions that, in turn, influence one’s functioning (Levy, 2009). These two above quotes from interviews reflect two approaches to a common experience. On the one hand, players accept the remarks from the bleachers as a natural and integral part of the game and the circumstances, something that they should respond to with restraint and even acceptance. On the other hand, according to the second quote, these remarks cause a serious emotional wound. As the findings show, the insult is especially biting because players expect their team’s fans to express support, which is why such remarks are considered to symbolize the fans’ betrayal of their role, and specifically their betrayal of the specific player in question. Although the fans’ remarks are received differently in these two approaches, all players are sharply aware of the taunts, curses, and remarks that come from the audience. It seems that players not only hear the taunts but are also directly affected by them (Epting et al., 2011).

Further, fans have the power to transform a player into an icon and to appropriate a player on behalf of a specific city; however, when a player exceeds the age of 30, his credit with fans diminishes and the spectators’ impatience with the player increases.

In his interview, Nimrod recounted that he feels very frustrated by the fact that, for the first time in his life, the people around him – the audience, media, and his family, ask him questions related to his retirement:

People suddenly ask me, “When are you going to retire?,” “What do want to do afterwards?,” “Does not anything hurt?,” “Isn’t there anything that bothers you?,” “Isn’t it difficult for you?.” All kinds of questions like that. People ask: “Are not you worried that the younger kids are trying to nip at your tail?.” Listen, it finds a mark! It seeps in. Let us say this, there’s no doubt about it, you suddenly start thinking about your career, about the next thing. It seeps in, mainly into your thoughts.

The direct remarks described by Nimrod and by Eran hurt the players and undermine their self-image. The audience’s behavior can be explained by the “role” that the audience appropriates for itself as a contributor to the team’s success and by its identification with the success of the player’s personal accomplishments and image (Tamir, 2022). This is reflected in fans’ efforts to sit in the bleachers and wear a player’s shirt, for example. A decline in a player’s performance as the player ages triggers counter-responses against what the audience believes is the player’s failure. Frequently, fans will wait for players outside their locker room, and after a bad game, fans might gang up on the player, curse him, and call him insulting names. Such incidents become embarrassing when the player is accompanied by family members. In this reality, dramaturgical demands of high-profile athletes who work in high publicity, visible contexts, become relentless and unremitting, as back-stage regions become fewer and further between (Roderick and Allen-Collinson, 2020). Such incidents and such taunts have a substantial impact on the player’s conduct, through their influence on the player’s emotional responses and self-image.

It appears that a player’s perceived age is directly related to the way the player is perceived by society. An athlete’s age is a critical factor in the management of their professional career, and at some point, players may become labeled by their age rather than by their personal accomplishments (Lally, 2007). The other aspect of a player’s stress, expectations, signification, and commercialization emerges when the audience becomes an obstacle in the player’s career, when the audience’s taunts and sarcastic remarks from the bleachers reduces the player’s time on the court. In rare cases, fans’ discontentment with a player might even lead to the player’s dismissal. In this way, the audience causes a double harm: First, to how players perceive themselves at this stage of their career, and second, the impact on professional issues that are reflected in the player and in the training crew (Stephan, 2003). In view of the dissonance between the athlete’s self-efficacy and the audience’s beliefs, the players feel as if they are receiving no support from the spectators who are ungrateful for the player’s past and current investments. Because the accepted view is that players are obligated to invest their talents and abilities into the game and fans are obligated to appreciate players’ efforts, players sense that such remarks fundamentally violate the formula and belittle their personal commitment. Such a feeling is not associated only with a specific point in time but seems to completely discount players’ enormous investments over the course of their career. Players expect recognition and respect, not only for their current performance but for the long-term contribution, which is also a symbol of their commitment to their club and their fans.

In summary, the findings are consistent with Hazan’s (2013) multifaceted definition of ageism, particularly in terms of highlighting the psychological sensitivity to the emotional consequences of exclusion. In this reference, it is evident that basketball players encounter prejudices and discrimination within the sports community, often facing stereotypes about their age and perceived physical decline. Nimrod’s statement, “It was very, very difficult. These were sharp, personal remarks and insults,” highlights a clear connection to the challenges that basketball players confront due to ageism. They endure emotional consequences when excluded from their once-inclusive environment, primarily because of their age, which significantly impacts their opportunities within the sports arena. This type of exclusion, particularly from fans who were once their staunch supporters, proves emotionally distressing for them.

### A farewell tour toward disengagement from one’s professional career

In contrast to those fans who express derision, as described above, others will express positive feelings for older players, whom they consider part of the team and whom they undoubtedly associate with the team’s history. For these fans, players are viewed as the soldiers who battle on behalf of the team and its fans, and many times become heroes (Levental and Nudelman, 2021). Although both types of response are driven by a recognition of a player’s older age, this recognition is expressed differently in both cases. For the second group of fans, curses and taunts are replaced by indications of admiration and respect. The relations between the audience and players follow unwritten rules related about each side’s commitments. Players are required to honor their fans and the audience through their performance on the court, and in exchange, players gain honor and support that position them as admired and revered members of the team. In view of this implicit contract, players feel that they cannot afford to disregard their fans even if the latter’s conduct borders on harassment, badgering, or behavior considered unjustified. Such fandom is not limited to the bleachers and occasionally manifests in the public sphere, outside sports arenas. It is important to note that while this type of relationship applies to all players, attitudes to older players change and manifest in ways that do not emerge with respect to younger players. Experienced basketball players sometimes become figures with whom fans love to identify, and frequently become a symbol that connects the team to its audience and to the media (Levental and Nudelman, 2021). In his interview, Shlomi, a 34-year-old basketball player focused on a description of the informal rewards that players might receive during their career:

I would go to entertainment spots…that respect you, if you can call it that…. Like, “come to us Shlomi … you go to some place… people clear the way for you. Great, come in, bring your friends” … some two or three guys show you respect, invite you. There were lots of places that naturally tried to attract you. It boosts your ego a little…it gives a kind of push to your ego.

Additional aspects of the inter-relations between players and the audience are reflected in statements by Dennis, a 39-year-old National Team player. He has played in Israel for over a decade, is married and has two children, and fans in Israel and Europe consider him to be one of the most loved and admired players in the league. He describes the relations between a player and the audience as follows:

I think the fans are the reason you play the game, you give back to them on the floor, you know – you play with your soul, and you see how much they care about the team. You just wanna give back to them, so yeah, I’m really connected. For me it comes easily – you want to give them the respect they give you, that’s why I’m always cozy with them. They want autographs, pictures, they see you on the street, you are driving in the car, they are waving… it’s really good to be loved.

Interviewees recounted that they often remain in the arena after a game to shakes fans’ hands, take photographs with them, and hand out autographs after other players leave the court. Many times, such practices will position older players as their team’s ambassador. Occasionally, players’ rich experience and history position them as team symbols even if they do not actually receive extensive game time. When older players come onto the court after a game has been effectively decided and there is no real significance to their presence, their entrance will be applauded by other players, the team staff, and especially the fans in the audience, who will greet the player warmly with praise and smiles that would not be doled out if he were younger and considered in his prime. Dennis stated that many times he would come onto the court with an apologetic smile, reflecting his discomfort at being in the spotlight. All he ever wanted was to shoot baskets and contribute to his team’s statistics, as expected of a young early-career player.

Dennis’ discomfort reflects the change in the natural relations between authentic fandom and players’ investments and contributions to their team. The give-and-take of this relationship is replaced by demonstrations of the fans’ respect for the player’s sports history rather than for the player’s current performance. In this reality, symbolic items such as souvenirs and merchandise related to a player often have significance. For example, despite Dennis’ age and his limited presence on the court, his uniforms were in extremely high demand because as team captain, he was the player most strongly identified with the team, its fighting spirit, and its loyalty to its fans. All in all, it seems that the interviewees acknowledge the fragility of this relationship and its reversibility. Players sought a balance between a desire to totally devote themselves to this relationship and their recognition that the relationship is a temporary one. The interviewees’ statements reflect the lack of trust they have in this uncertain relationship, which wavers between love and hate between a player and the fans over the course of his career. Also evident is the older players’ resignation and acceptance of the loss of their glory and fame (Jewett et al., 2019).

One case that attests to this nature of the player-audience relationship was a decisive game between Hapoel Tel Aviv and Maccabi Tel Aviv in the 2022/23 season. In this game, when the score had already been decided (often referred to as “garbage time”), Guy Pnini walked onto the court for the first time in that game. Guy is the former Maccabi Tel Aviv captain and one of the team’s most senior Israeli players in recent history. His participation in the game was not based on professional considerations but motivated by a desire to show him respect and give fans an opportunity to demonstrate their admiration and respect for him. Although the situation was obvious to all and is a well-known practice in the world of sports, it structures the specific social roles within the player-audience relationship. The audience is required to honor the player, but not for the performance, while the player understands that the audience’s adulation is granted for other periods of his career. The player senses that the final chord in their career is approaching, but, as the interviewees noted, the player typically believes that a summary of their career is premature.

### The media’s role in shaping beliefs and attitudes

The two themes noted above are related to fan behavior toward older players. A question arises, however, regarding the process through which fans develop their attitudes toward the players. When do fans develop a belief that the retirement of an older player is approaching? Although this may be inferred from the length of a player’s time in the game (based on the coach’s professional considerations) or a decline in the player’s performance (based on subjective and quantifiable measures), the findings of the current study confirm that the media play a decisive role in shaping fans’ options, in line with findings of previous studies (e.g., Phua, 2010). As articulated by McCombs (2004, 2005), the media not only inform us about what to think but also guide us in how to think about it. Specific attributes can strike a chord with the public to the extent that they evolve into particularly persuasive arguments for emphasizing the importance of the subject, individual, or topic in question. Within the sports domain, the rhetorical framing of athletes prominently depends on age as a representation of experience, as highlighted by Carmack and Lazenby (2023).

One example of how the media use stereotypes to construct a player’s profile, and to a large extent also the character of a team, occurred during a season hiatus, when the daily newspaper Ha’aretz published an article about Maccabi Ashdod, a team with two senior players over the age of 30, under the headline “Let the Veterans Play Before Us” (Ha’aretz Sports, July 5, 2011). The headline is a paraphrase of the well-known biblical phrase, “Let the young men arise and play before us,” which expresses a disparaging attitude toward inexperienced people who try to engage in practices in which they are unskilled. Attributing the image of incompetency to the team’s older players implies that they are no longer able to perform in the tough league due to their age, and are no longer able to move, shoot baskets, or perform other roles that require dexterity, concentration, and physical contact, because their bodies are no longer young or agile. According to this article, “The team needs a mix of young people and responsible adults. Ashdod would be wise to put some new blood next to the older ones.” In the eyes of the media, if the team wishes to be successful it should sign young players, this would help the older players maintain their relevance and contribute added value to the team, and they would become the “responsible adults.” Another example that reproduces the “old player stereotype” is the question posed in the secondary headline: “Can a reduced ego and extensive experience compensate for a weakening body?.” In this manner, the media structure players’ retirement around stereotypes related to age and physical decline, which are framed as the sole cause of retirement from basketball (Vieira and Sousa, 2020).

The interviewees in the current study describe a similar picture regarding the media’s role as a consciousness-shaping mechanism. All interviewees acknowledge the media’s power, their practices, and their use of rhetoric, and mentioned specific cases in which the media criticized them, specifically referencing their age. Yossi, an experienced agent explained:

I talked to a player, and he says, “Tell me, how can that son of a bitch [from the media] say that I’m done? Come on, I still do 12 min in a game, did not you see how I guarded him?... It’s enough for two people from the media to say it for everyone to follow them… it has an effect on [other] teams’ views..” All day they think: “Enough, he’s at the end of his game. He cannot move his feet, enough, stop, stop, stop...so then you are considered a player who’s over the hill, and your financial offers will be accordingly..”

Shlomi offered a similar description:

The media…every time they broadcast a game, they talk about your performances, “He’s an old man, he’s old, he is not what he used to be….” Look, it’s natural that they try to push the young ones and look for the next stars… the next big thing… but somehow in that period, which is quality time for players… and they are at the peak of their performance mentally, and also physically, I think, they are at a very high standard at 30–32. Their physical [condition] is ideal. You know, [at that age] you know your body best of anyone, you know how to take care of it, you know how to train, you know when to stop training, you are in the best place you can be.

The interviewees expressed their frustration for having the label of “aging basketball player” attached to them. Labeling affects the way a player’s image is presented to media viewers and readers (Hasaan et al., 2016). A uni-dimensional presentation of a player’s age in bureaucratic and stereotypical terms defeats the player who then succumbs to this image of an “aging player” in the retirement phase. A decision to retire against the player’s own personal wishes indicates that the player has succumbed to the image that the media constructed.

The tendency to criticize active players is reversed when a player announces their retirement. In Dennis’ case, for example, when he announced his decision to become a coach, the media’s response was generous and kind, exalting his achievements. The media’s support for this announcement illustrates its position as a key player in creating a player’s public image. After the media satisfied its own needs, they were able to glorify Dennis’ past and build up his image, catapulting him toward a new, more “respectful” and age-appropriate career. The following quote is taken from one of the dozen articles related to Dennis’ extensively covered retirement announcement:

One looks at [his] list of achievements and it is obvious to us all that he is a star. Perhaps the only one who enjoys a consensus of love, including from his colleagues…his sacrifice, fighting spirit, winner mentality, talent, love of the game, and above all, enormous modesty. That’s the formula that the man we know as Dennis is made of (Ynet Sport, July 22, 2011).

After a player retires, criticism and the focus on their aging body and advanced age transform into a slew of superlatives, support, and positive media coverage. From an oddity, the player suddenly becomes a star with heroic traits. Nonetheless, Dennis was relegated to public memory, he was “buried” in a glorified manner, is spoken of in the past tense, and the commemoration of his accomplishments became the material for what Hall and Gieben call “memory work” to build the self and its memory. These are the raw materials used in the construction of the player’s self-image. Through a retrospective review of the player’s achievements, the media establishes a new “self” for the player, one that is consistent with the social order, and these ultimately affect the self-image of the retiring player.

Self-image is a set of beliefs and opinions about oneself, one’s values, competencies, and social status. It is one of the main factors that affects a person’s behavior. Self-image is dynamic, and is affected by changes in a person’s physical, mental, social, and cognitive condition. Lev (2020) argued that self-image is a product of the athlete’s environment, which is the source of the experiences necessary for a person’s development; not only to be human but also to feel human, as a connected energized member of the sport community (Lev, 2022). As a significant socialization agent, the media also directly affect the player as a media consumer, and indirectly affect the player’s self-image by shaping public opinion. As the findings enable us to discern, the media frame older basketball players as too old to continue playing professional sport by underscoring their age as a central point of discussion. Moreover, the media is the body that sets professional sports within chronological age boundaries and does not necessarily make professional assessments of situations (van Sterkenburg et al., 2010). It affects the audience, and the audience influences the beliefs and opinions of the professional circles that affect players, including their training teams, the audience, and the media. This framing serves to diminish their accomplishments, reducing them to mere statistics that might suggest they no longer belong in their respective sports (Carmack and Lazenby, 2023). In light of this reality, it is unsurprising that both players and fans are influenced by the nature of the media coverage. It is suggested that all of these factors implant in players a specific idea about their abilities, even if this idea conflicts with their own embodied sensations and perceptions, and they play a significant role in their retirement decision.

## Conclusions and summary

Researchers’ attention to elite athletes’ retirement has typically focused on the athletes themselves and the process they experience on their path to retirement. Studies have also examined the professional, emotional, financial, and other challenges that athletes encounter in adapting to their new situation. The current study focuses on the fans’ role and their contribution to the retirement decision. Sports fans are considered a significant factor in modern sports, and this is certainly the case in popular fields of sport where their impact on the sport in general, and specifically on the players, is dramatic.

This study offers a new perspective on the audience’s role in shaping athletes’ retirement trajectories. Findings point to three main themes: the media’s influence on public opinion and public discourse; the audience’s effect on athletes; and the athletes’ self-image development process through the compliments and taunts directed at them. These themes shed light on a process that has thus far gained limited attention in academic and professional literature. Research on fans’ behaviors and impact are typically examined at the team level, with respect to the team’s accomplishments. The novelty of the current research is its analysis of the audience’s effect on athletes’ attitudes and beliefs and how these shape retirement-related decisions. The findings of the current study indicate that the audience does not merely function as a backdrop for athletes’ performances, but has the power, at the collective and the individual level, to affect change on and off the court. Significantly, the findings emphasize the challenges that players must confront within an ageist environment. In this context, it becomes evident that the aging process of professional basketball players is, to a great extent, influenced by social constructs.

The current study offers a preliminary focused examination of the phenomenon under investigation. Participants are all elite basketball players with a history in Israel’s premier league. It is therefore important to examine the effect of the audience’s conduct on the retirement of players in other fields. Due to cultural and social differences related to the audience’s size and conduct, future studies on additional fields or sport and levels of competition can help shed light on the differences and similarities of these cases. Furthermore, attention should also be directed to gender and cultural factors and to how the social and cultural environment in which athletes and the audience function affect the dynamics of their interrelationship.

## Data availability statement

The original contributions presented in the study are included in the article/supplementary material, further inquiries can be directed to the corresponding author.

## Ethics statement

The study was conducted in accordance with all ethical standards set by Haifa University, Israel, and received approval from the Ethical Committee for Research Studies in Master’s Degree programs. The studies were conducted in accordance with the local legislation and institutional requirements. The participants provided their written informed consent to participate in this study.

## Author contributions

AL: Conceptualization, Data curation, Formal analysis, Investigation, Methodology, Project administration, Supervision, Validation, Writing – original draft, Writing – review & editing. OL: Conceptualization, Formal analysis, Writing – original draft, Writing – review & editing. IT: Conceptualization, Formal analysis, Writing – original draft, Writing – review & editing.
